# Prevalence and Risk Factors of Sleep Disordered Breathing in Fabry disease: A Prospective Cohort Study

**DOI:** 10.1097/MD.0000000000002413

**Published:** 2015-12-31

**Authors:** Daniel Franzen, Nicolas Gerard, Daniel J. Bratton, Annette Wons, Thomas Gaisl, Noriane A. Sievi, Christian F. Clarenbach, Malcolm Kohler, Pierre A. Krayenbühl

**Affiliations:** From the Division of Pulmonology, University Hospital Zurich, Raemistrasse, Zurich (DF, NG, DJB, AW, TG, NAS, CFC, MK); and Department of Internal Medicine, Regional Hospital Linth, Gasterstrasse, Uznach, Switzerland (PAK).

## Abstract

Excessive daytime sleepiness (EDS) is a frequently reported and not well-understood symptom in patients with Fabry disease (FD). Sleep-disordered breathing (SDB) is a possible factor. As deposition of glycosphingolipids in the upper airway muscles is likely, we hypothesized that obstructive sleep apnoea (OSA) is highly prevalent in FD and positively associated with its severity.

All patients with FD who are followed in the Fabry cohort of the University Hospital Zurich (n = 62) were asked to participate in this prospective cohort study. Eligible patients were prospectively investigated by assessing their daytime sleepiness using the Epworth Sleepiness Scale (ESS), the severity of FD using the Mainz Severity Score Index (MSSI), and by an ambulatory overnight respiratory polygraphy between November 1, 2013, and January 31, 2015. SDB was defined as an apnea/hypopnea index (AHI) of > 5/h.

Fifty-two patients (mean ± SD age 42.8 ± 14.7 years, 33% men, mean ± SD BMI 23.4 ± 3.6 kg/m^2^) with a median (IQR) MSSI of 12 (5–19) were included. Median (IQR) ESS was 6 (2–10) and 7 patients (14%) had an ESS > 10. Thirteen patients (25%) had SDB (78% obstructive sleep apnea, 22% central sleep apnea). In the multivariable analysis, the age was the only statistically significant predictor of SDB (OR 1.11, 95% CI 1.04–1.18, *P* = 0.001). ESS was associated with depression (*P* < 0.001) but not AHI nor age.

This study shows that SDB, especially obstructive sleep apnea is highly prevalent in patients with Fabry disease. However, EDS in FD seems to be related with depression rather than SDB.

ClinicalTrials.gov (identifier: NCT01947634).

## INTRODUCTION

Fabry disease (FD) is an X-linked lysosomal storage disease leading to multiorgan dysfunction due to deposition of glycosphingolipids in various tissues.^1^ Excessive daytime sleepiness (EDS) is a frequent and early reported symptom in patients with Fabry disease, which has a major impact on their quality of life.^2^ In a cohort of 49 patients (27 men; mean age 43 years) with genetically proven Fabry disease, the prevalence of EDS was 68% exceeding most other symptoms related to FD.^2^ By contrast, the prevalence of EDS in the adult European population is 18%.^3^ The cause of EDS in FD is not well understood. On one hand, chronic renal or cardiac dysfunction irrespective of the underlying cause may lead to chronic fatigue. However, it has been shown that chronic fatigue appears in Fabry patients without renal or cardiac dysfunction.^4^ And, fatigue must be distinguished from EDS, which is defined as the inability to maintain wakefulness and alertness during the major waking episodes of the day, with sleep occurring unintentionally or at inappropriate times almost daily for at least 3 months,^5^ whereas fatigue rather refers to a subjective lack of physical or mental energy. Eventually, patients may use terms such as fatigue and sleepiness interchangeably, and data from patients with sleep disorders suggest that the terms have significant overlap.^6^ Sleep-disordered breathing (SDB) defined as obstructive (OSA) or central sleep apnea/hypopnea (CSA), the latter including Cheyne–Stokes respiration, may be the reason or at least a major contributing factor for EDS in Fabry patients. In this regard, in 1 case-control study of 23 patients with FD, overnight polysomnography revealed Cheyne–Stokes respiration in 22% of subjects.^7^ Further, brain magnetic resonance tomography showed confluent white matter lesions including the brain stem.^7^ As previous studies have demonstrated an association between white matter lesions and SDB,^8,9^ some authors suspect an association of the white matter lesions detected in their patients and the Cheyne–Stokes respiration most likely leading to EDS.^2,7^ Their conclusion is supported by the fact that severe progressive cerebral white matter lesions are common findings in Fabry disease, occurring early in the disease process and worsen with time.^10,11^ However, Cheyne–Stokes respiration cannot be related to cerebral white matter lesions alone as it requires an increased circulation time, usually caused by cardiac failure.^5^ Eventually, both entities can be present in Fabry patients. As accumulation of glycosphingolipids in the upper airway muscles is also likely, we hypothesized that OSA is also highly prevalent in FD and is associated with its severity.

## METHODS

### Subjects

**FIGURE 1:**
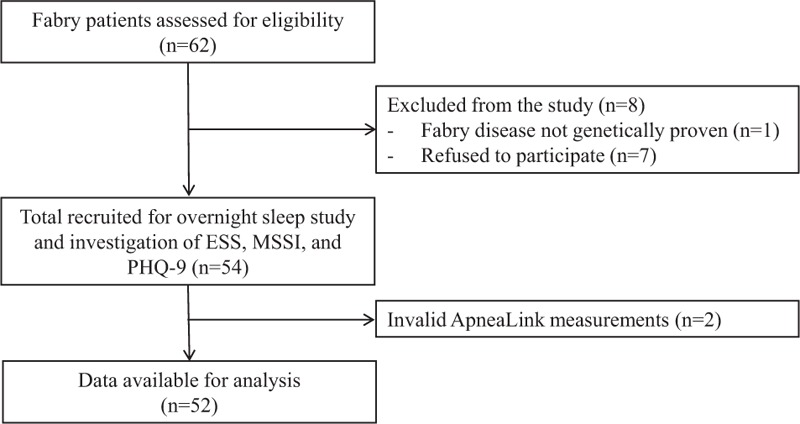
Study flowchart. ESS = Epworth sleepiness scale; MSSI = Mainz Severity Score Index; PHQ-9 = patient health questionnaire.

All patients with genetically proven FD above the age of 18 years and who are followed in the Fabry cohort of the University Hospital Zurich (n = 62) were asked to participate in the study (Figure 1). Written informed consent was obtained from all patients included in the study. The study was approved by the Ethics Committee of the Canton of Zurich, Switzerland (KEK-ZH 2013–153), and is registered at ClinicalTrials.gov (identifier: NCT01947634). This study was conducted according to the STROBE statement.

### Study Intervention

In this cross-sectional observational study, all eligible patients were investigated by performing an unattended sleep study at home with 1 overnight respiratory polygraphy using an ApneaLink plus (ResMed Ltd, Bella Vista NSW, Australia) device. The device records 5 channels of information allowing the differentiation between obstructive and central apneas during sleep: the patient's nasal respiratory flow signal, respiratory effort, pulse, snoring, and transcutaneous oxygen saturation. It has been validated as an accurate instrument to detect apneas/hypopneas and oxygen desaturations.^12,13^ The results of the overnight examination were scored manually according to the standard criteria of the American Academy of Sleep Medicine ^5^ by 2 investigators (AW and TG) who were blinded to the patients and their physical data. Apneas were defined as a cessation of airflow lasting >10 s and hypopneas as a reduction of airflow of at least 30% lasting >10 s, associated with a fall in oxygen saturation of >3%.^5^ SDB was defined as an apnea/hypopnea index (AHI) of >5 events/h. Examinations took place between November 1, 2013, and January 31, 2015. The patient's daytime sleepiness was assessed using the German version of the Epworth Sleepiness Scale (ESS).^14,15^ The ESS is a self-administered questionnaire to quantitatively measure sleep propensity during normal daily activities and can be helpful to distinguish true EDS from fatigue or low energy.^14^ An ESS > 10 was considered to indicate clinically relevant EDS. The severity of FD was determined using the Mainz Severity Score Index (MSSI). The MSSI is a clinical scoring system considering general, neurological, cardiovascular, and renal abnormalities, which classifies disease burden by 3 grades (mild < 20, moderate 20–40, severe > 40).^16^ Specific organ involvement of FD was defined according to MSSI.^16^ Central nervous system (CNS) involvement included history of stroke or transient ischemic attack and/or ischemic lesions on magnetic resonance imaging (MRI) or CT. Cardiovascular involvement was defined as clinical or echocardiographic signs of congestive heart failure and/or increased levels of N-terminal pro-B-type natriuretic peptide (NT-proBNP) and/or changes in cardiac muscle thickness on echocardiography or MRI and/or valve insufficiency. Age- and sex-specific cutoff values of NT-proBNP were considered to indicate congestive heart failure as the dichotomous variable. The reference values were as follows: (males/females): < 45 years (85.8/130 ng/L); 45 to 54 years (121/249 ng/L); 55 to 64 years (210/287 ng/L). Renal affection was present if the glomerular filtration rate was <60 mL/min and/or there was evidence of proteinuria. Depression was assessed using the self-administered patient health questionnaire-9 (PHQ-9), which has been found to have acceptable diagnostic properties for detecting a depressive disorder for cutoff scores between 8 and 11.^17^

### Outcome Measures

The primary outcome was the prevalence of EDS and SDB, the latter classified as OSA or CSA. The secondary outcome was to investigate possible risk factors of SDB and ESS. Independent variables included sex, age, body mass index (BMI), neck circumference, CNS, cardiovascular or renal involvement of Fabry disease, MSSI, ESS, forced vital capacity (FVC), forced expiratory volume in 1 s (FEV1), left ventricular ejection fraction (LV-EF) and left atrial diameter (LAD) on transthoracic echocardiography, NT-proBNP, and PHQ-9.

### Statistical Analysis

All statistical analyses were performed using Stata version 14 (College Station, TX: StataCorp LP). Data are summarized as median (interquartile range [IQR]), mean ± standard deviation (SD), or percentages as appropriate. The distribution of continuous variables was assessed using the skew statistic and normal quantile plots. Differences between patients with and without SDB were assessed using χ^2^ tests for binary outcomes and Mann–Whitney *U* test or unpaired *t* tests for continuous outcomes as appropriate. The individual association between SDB and each potential risk factor listed above was initially assessed using univariable logistic regression analysis. A multivariable logistic regression model using a backward selection procedure with *P* value for removal of 0.1 was then used to find independent predictors of SDB. A similar procedure using linear regression was used to assess associations with ESS. All tests were 2-sided with a *P* value <0.05 considered to indicate statistical significance.

## RESULTS

**FIGURE 2:**
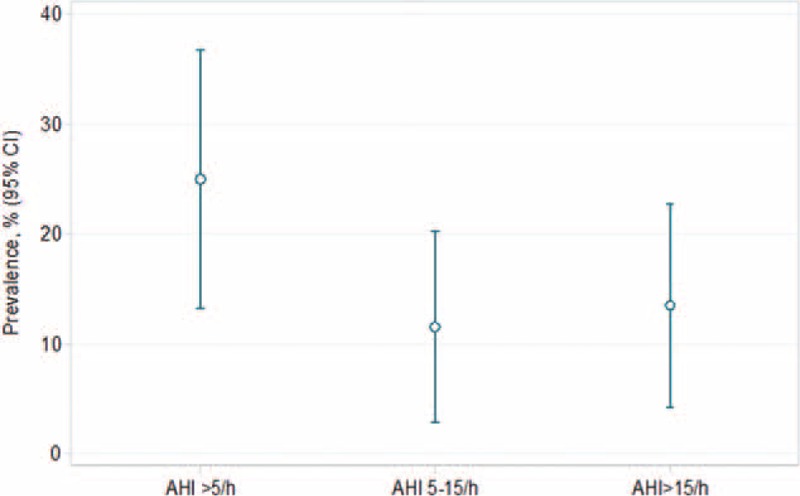
Prevalence of sleep-disordered breathing in the Fabry cohort (n = 52). AHI  = apnea/hypopnea index.

From the 62 patients in the Fabry cohort, 2 patients were excluded because of invalid ApneaLink measurements. Another patient was excluded because FD was not genetically proven, and 7 patients refused to participate in the study. The remaining 52 patients were included in the study. Patient characteristics are presented in Table 1. Seven participants had an ESS > 10 (prevalence of EDS 14%, 95% CI 4, 24%). The results of the overnight sleep examination are displayed in Table 2. The prevalence of SDB was 25% (95% CI 13, 37%). Of the 13 patients with SDB (78% OSA), 6 patients (12%) had mild SDB (AHI between 5 and 15) and 7 (13%) had moderate to severe SDB (AHI > 15) (Figure 2).

**TABLE 1:**
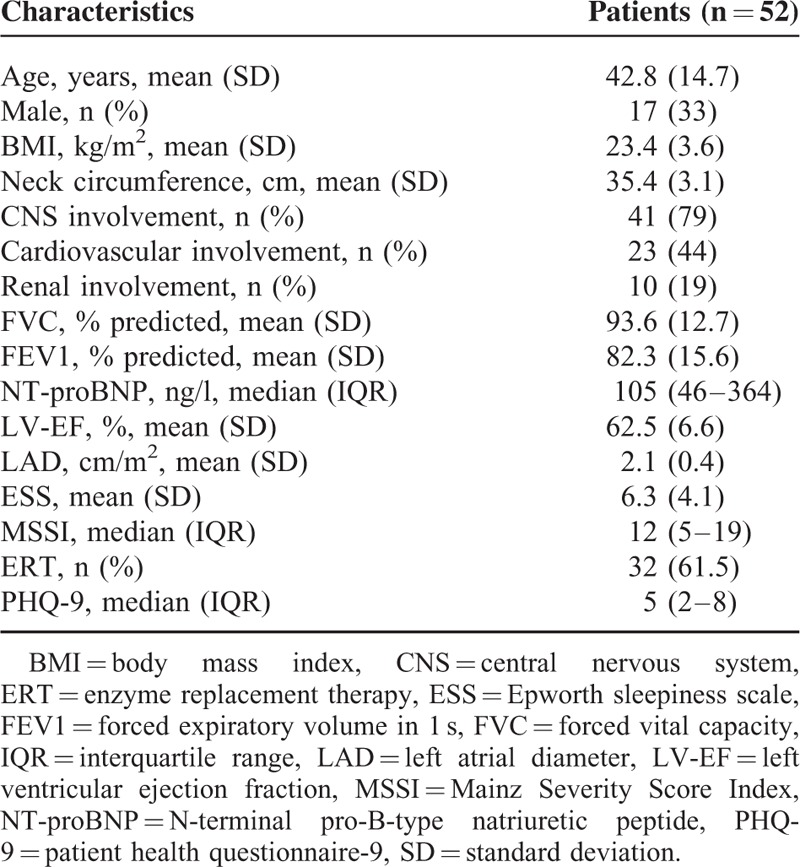
Patient Characteristics

**TABLE 2:**
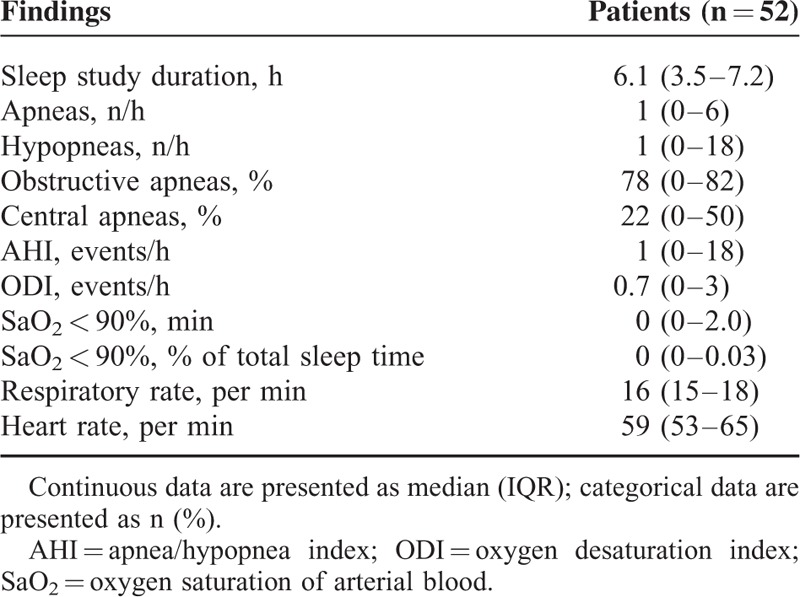
Results of the Overnight Respiratory Polygraphy

Compared to patients with an AHI ≤ 5, subjects with SDB presented higher age, BMI, MSSI, NT-proBNP, and LAD, and cardiovascular involvement of FD (Table 3). ESS was comparable in patients with and without SDB. In univariable analyses, age (*P* = 0.001), cardiovascular involvement of FD (*P* = 0.01), NT-proBNP (*P* = 0.008), LAD (*P* = 0.009), and MSSI (*P* = 0.042) were significantly associated with an increased odds of SDB (Table 4). However, in the multivariable regression model with backward selection only age remained. The number of central apneas per hour during sleep was not correlated with either LAD (*r* = 0.12; *P* = 0.44) or proBNP (*r* = −0.07; *P* = 0.64). The 4 covariates which were highly significant in the univariable analyses (age, NT-proBNP, LAD, and cardiovascular involvement) were correlated with each other.

**TABLE 3:**
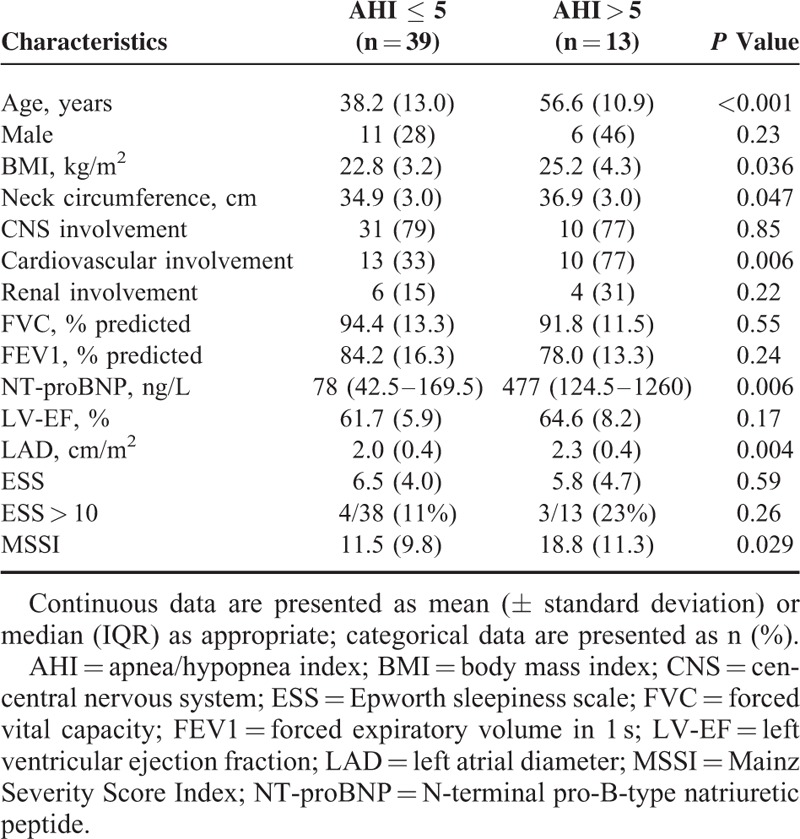
Patient Characteristics Classified into Patients Without and With OSA

**TABLE 4:**
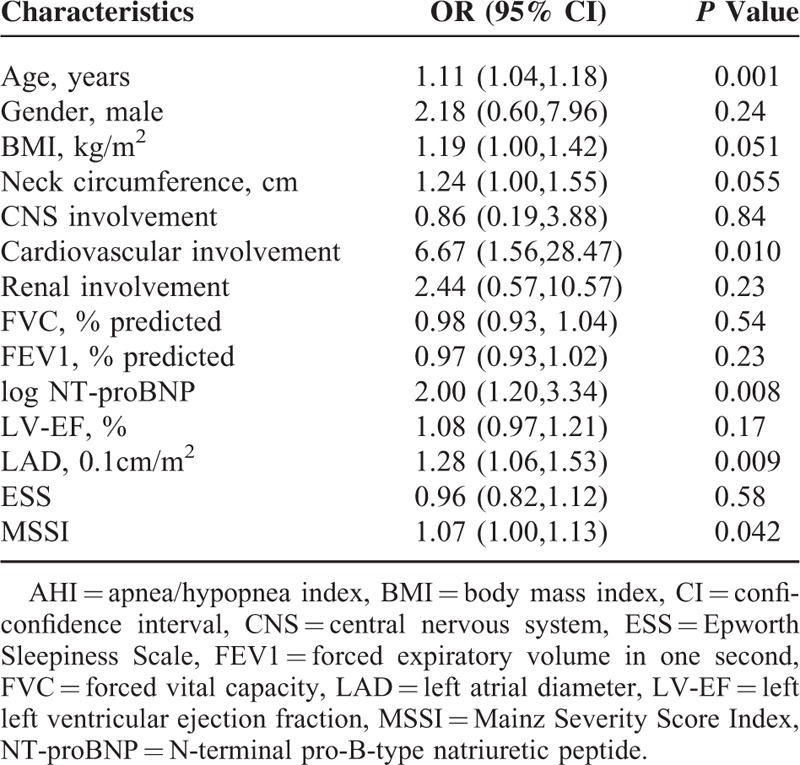
Univariable Logistic Regression Analysis of Factors With a Possible Association With AHI > 5

Notably, AHI was not significantly associated with ESS (*P* = 0.18). Instead, MSSI (*P* = 0.005), CNS involvement (*P* < 0.001), and PHQ-9 (*P* < 0.001) were significantly associated with ESS in univariable regression analyses. In the multivariable regression analysis with backward selection, only PHQ-9 (β=0.5, 95%CI 0.2–0.7, *P* < 0.001) and CNS involvement (β=2.8, 95%CI 0.5–5.2, *P* = 0.018) were statistically significantly associated with ESS (Table 5).

**TABLE 5:**
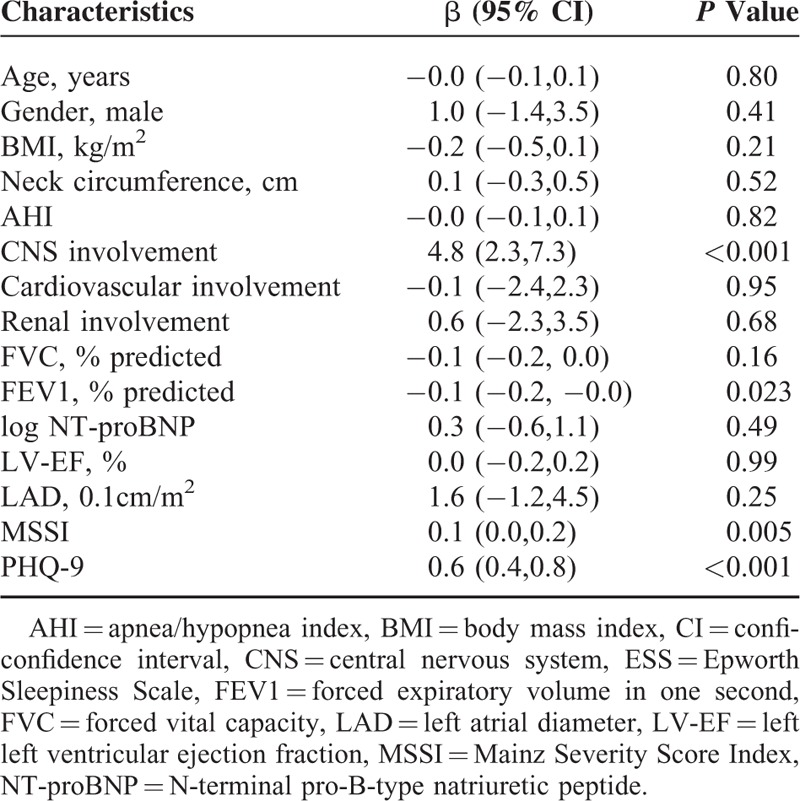
Univariable Linear Regression Analysis of Factors With a Possible Association With ESS

## DISCUSSION

The aim of this study was to investigate the prevalence of SDB and EDS in a cohort of patients with genetically proven Fabry disease. We hypothesized that sleepiness, which has been shown to be highly prevalent in these patients,^2^ is possibly induced by SDB among other reasons. Furthermore, we aimed to detect predictors for SDB and EDS in these patients. To the best of our knowledge, this is the first study showing such a high prevalence of SDB, especially OSA, in patients with Fabry disease. The prevalence of SDB in the investigated Fabry patients (25%) was substantially higher than that in the general adult European population, in which the reported prevalence of SDB ranges between 3% and 19% in men and 2% and 15% in women.^18–21^ Considering a matched American cohort, Peppard et al found a prevalence of mild to severe SDB (defined as AHI > 5) ranging between 1% and 18% in 30 to 49 years old subjects with a BMI < 25.^22^ However, it has to be mentioned that the prevalence of OSA is differing globally, depending on the geographical region.^21^ Therefore, our findings may not be generalized to other regions. In a recent study on 23 Fabry patients performed by Duning et al, the prevalence of SDB was 22%.^7^ In contrast to our study, Duning et al confined SDB to CSA with Cheyne–Stokes respiration rather than OSA, which has been attributed to microstructural changes in the brainstem. However, a causative relationship between these reported white matter lesions and SDB is speculative, since all of the Fabry patients without CSA also have analog changes on MRI.^7^ Furthermore, cerebral white matter has been shown to be extensively affected in OSA patients without FD.^23,24^ Hypothetically, the reported white matter lesions in patients with FD could also be due to OSA, and therefore these lesions may account for the development of CSA. According to our own (unpublished) data, we found that 50% of Fabry patients had cerebral white matter lesions which were consistent with the findings reported by Reisin et al.^25^ However, these changes were not significantly associated with SDB.

Only a seventh of the patients were considered to have EDS using a threshold of ESS > 10 suggesting that ESS may not be the adequate tool to measure sleepiness in patients with FD. Using the ESS, the prevalence of EDS in our cohort is comparable to the one in the general adult European population.^3^ In the multivariable analysis, only depression (PHQ-9) and CNS involvement by FD were significantly associated with ESS. However, at least the association of depression and ESS is not new, as ESS is a subjective measurement with the tendency to over-report unrefreshed sleep and daytime somnolence. Anyway, OSA and depression have overlapping symptoms, thereby resulting in a complex relationship.^26^

Generally, risk factors for OSA include obesity, upper airway abnormalities, male sex, and age.^27,28^ In the present cohort with nonobese (median BMI 23 kg/m^2^) and comparably young Fabry patients with a mean age of 43 years, only age was associated with SDB suggesting the existence of other risk factors for SDB in FD. However, due to lack of power we possibly have not found them. As storage of globotriaosylceramide in smooth muscle cells seems to play a key role in the pathogenesis of myocardial dysfunction and bronchial involvement in FD,^29,30^ we hypothesized an analog mechanism in the upper airway muscles, which may contribute to the development of OSA. Hence, MSSI, a measure of the severity of FD, could therefore be an indicator of this proposed mechanism. However, MSSI was only associated with SDB in the univariable analysis. Interestingly, age was associated with higher MSSI—therefore the risk of OSA may be higher in older FD patients because of more severe disease or the prolonged exposure to the disease. In the end, the present analysis of data does not definitively prove this hypothesis. Another interesting hypothesis regarding the pathogenesis of SDB in FD is REM-sleep associated hypoventilation, which is typically seen in patients with neuromuscular disease.^31^ A deposition of glycosphingolipids in respiratory muscles, mainly in the diaphragm possibly results in a propensity to nocturnal hypoventilation. Unfortunately, the overnight respiratory polygraphy instrument used in our study is inadequate to detect hypoventilation, and blood gases nor transcutaneous carbon dioxide were measured for purpose of the study. Therefore, we cannot comment on this also possible mechanism. However, FVC, which is normally reduced in patients with a relevant neuromuscular disease, was not associated with SDB nor ESS. And, considering the small sample size in our study, the significance level of FEV1 is possibly too low to represent a meaningful association with ESS.

In univariable testing, NT-proBNP and LAD, both markers of the hemodynamic severity of heart failure, were associated with the presence of SDB in Fabry patients. In patients with end-stage renal disease not confined to Fabry disease, a relationship between left atrial size and OSA severity has been described recently.^32^ In addition, left atrial size has been shown to be a predictor of CSA in a study on 62 non-Fabry patients with chronic heart failure.^33^ However, MSSI, NT-proBNP, and LAD did not remain statistically significant in the multivariable analysis. And, the number of central apneas was not correlated with either LAD or proBNP. Apart from age we were not able to identify any other independent predictors of SDB in the present cohort of Fabry patients. This leaves the question open as to whether a possible accumulation of glycosphingolipids is directly responsible for the development of OSA and whether Fabry-related organ dysfunction may also contribute. Nonetheless, cardiovascular involvement of FD was also associated with SDB in a univariable analysis. As OSA has various effects on cardiovascular damage in the general population,^34^ a possibly potentiating effect of OSA on cardiovascular morbidity might also be expected in patients with FD and requires further investigation.

There are some limitations concerning the present results, which are primarily due to the design of a cross-sectional study without control group. The number of participants is likely to be too low to identify various independent predictors of SDB. Considering the rare occurrence of Fabry disease, we think that both issues could be addressed in a multicenter study. Finally, SDB was not investigated using polysomnography as the gold standard for overnight sleep studies. However, ApneaLink plus is sufficiently validated for the purpose of diagnosing SDB excluding REM-sleep-associated hypoventilation.^12,13^

## CONCLUSION

This is the first study showing that SDB, especially OSA, is highly prevalent in patients with Fabry disease. ESS was shown to be associated with PHQ-9 and CNS involvement but not AHI. We identified age as an independent predictor of OSA in the present cohort, leaving the question open of whether a possible accumulation of glycosphingolipids is the reason for the development of OSA and whether Fabry-related cardiovascular disease may also contribute. Although we found a significant correlation between age and the severity of FD (MSSI), we were not able to detect a direct association between MSSI and SDB. However, a potentiating effect of OSA on cardiovascular morbidity in patients with FD should be addressed in a long-term prospective study.
